# PERK signaling through C/EBPδ contributes to ER stress-induced expression of immunomodulatory and tumor promoting chemokines by cancer cells

**DOI:** 10.1038/s41419-021-04318-y

**Published:** 2021-11-01

**Authors:** Namratha Sheshadri, Dipak K. Poria, Shikha Sharan, Ying Hu, Chunhua Yan, Vishal N. Koparde, Kuppusamy Balamurugan, Esta Sterneck

**Affiliations:** 1 Laboratory of Cell and Developmental Signaling, Center for Cancer Research, National Cancer Institute, 1050 Boyles Street, Frederick, MD USA; 2 Center for Biomedical Informatics and Information Technology, Center for Cancer Research, National Cancer Institute, 9609 Medical Center Dr., Rockville, MD USA; 3 CCR Collaborative Bioinformatics Resource (CCBR), Center for Cancer Research, National Cancer Institute, Bethesda, MD USA; 4 Advanced Biomedical Computational Science, Frederick National Laboratory for Cancer Research, Frederick, MD USA; 5 Present Address: Susan Lehman Cullman Laboratory for Cancer Research, Ernest Mario School of Pharmacy, Rutgers, The State University of New Jersey, 164 Frelinghuysen Road, Piscataway, NJ 08854-8020 USA

**Keywords:** Stress signalling, Cancer microenvironment, Transcription

## Abstract

Cancer cells experience endoplasmic reticulum (ER) stress due to activated oncogenes and conditions of nutrient deprivation and hypoxia. The ensuing unfolded protein response (UPR) is executed by ATF6, IRE1 and PERK pathways. Adaptation to mild ER stress promotes tumor cell survival and aggressiveness. Unmitigated ER stress, however, will result in cell death and is a potential avenue for cancer therapies. Because of this yin-yang nature of ER stress, it is imperative that we fully understand the mechanisms and dynamics of the UPR and its contribution to the complexity of tumor biology. The PERK pathway inhibits global protein synthesis while allowing translation of specific mRNAs, such as the ATF4 transcription factor. Using thapsigargin and tunicamycin to induce acute ER stress, we identified the transcription factor C/EBPδ (*CEBPD*) as a mediator of PERK signaling to secretion of tumor promoting chemokines. In melanoma and breast cancer cell lines, PERK mediated early induction of C/EBPδ through ATF4-independent pathways that involved at least in part Janus kinases and the STAT3 transcription factor. Transcriptional profiling revealed that C/EBPδ contributed to 20% of thapsigargin response genes including chaperones, components of ER-associated degradation, and apoptosis inhibitors. In addition, C/EBPδ supported the expression of the chemokines CXCL8 (IL-8) and CCL20, which are known for their tumor promoting and immunosuppressive properties. With a paradigm of short-term exposure to thapsigargin, which was sufficient to trigger prolonged activation of the UPR in cancer cells, we found that conditioned media from such cells induced cytokine expression in myeloid cells. In addition, activation of the CXCL8 receptor CXCR1 during thapsigargin exposure supported subsequent sphere formation by cancer cells. Taken together, these investigations elucidated a novel mechanism of ER stress-induced transmissible signals in tumor cells that may be particularly relevant in the context of pharmacological interventions.

## Introduction

The endoplasmic reticulum (ER) is the primary site for synthesis, folding and post-translational processing of proteins. Cancer cells experience chronic ER stress due to intracellular activation of oncogenes and loss of tumor suppressors as well as microenvironmental stressors such as nutrient deprivation and hypoxia [1]. The ensuing unfolded protein response (UPR) is a cytoprotective mechanism to alleviate stress and restore cellular homeostasis [2]. The ER stress sensors ATF6, IRE1 and PERK trigger transcriptional reprogramming mediated by ATF6-N, XBP1S and ATF4, respectively, to effect the stress response pathways [3–5]. Cancer cells hijack these pathways to promote cell survival under stress and thereby facilitate tumor metastasis and therapy resistance [6, 7]. However, prolonged, unmitigated stress tips the balance toward apoptotic pathways [8]. Cancer cells that exhibit already elevated basal ER stress signaling can be hypersensitive to further ER stress, a concept that is being explored for cancer therapy [9]. For example, thapsigargin, the active component of the pro-drug mipsagargin, which is in clinical trials for advanced refractory cancers of the breast, brain, prostate and liver, is an inhibitor of the SERCA pump and causes ER stress through loss of calcium from the ER [10, 11].

Of the three UPR sensors, PERK inhibits global protein synthesis through phosphorylation of eIF2α, which in turn triggers the synthesis of specific proteins such as the ATF4 transcription factor. ATF4 mediates many adaptive responses but also induces the CCAAT/enhancer binding protein (C/EBP) homologous protein (CHOP, encoded by *DDIT3*), which can redirect the response toward cell death. PERK also collaborates with IRE1 to activate the NF-κB transcription factor, which mediates the expression of pro-inflammatory cytokines [12, 13]. Thus, in addition to cancer cell intrinsic cell survival and death pathways, the ER stress-induced secretion of cytokines contributes to the transmission of ER stress and inflammation [12, 14]. Given the Janus-faced nature of ER stress in the context of cancer, it is important to fully decipher the various UPR pathways and their contributions to tumor biology.

The transcription factor C/EBPδ (*CEBPD*) is a modular regulator of diverse cell signaling pathways that control cell proliferation, differentiation, survival or death, depending on cell type and context [15]. C/EBPδ expression is typically low, but induced in response to stimuli that include inflammation, hypoxia, and DNA damage [15], conditions that are known to induce ER stress. Accordingly, *Cebpd*-deficient mice are hypersensitive to high dose bacteremia and ionizing radiation [16, 17]. In breast cancer cells, C/EBPδ promotes stemness through amplification of hypoxia and IL-6 signaling [18]. Here, we report that under acute ER stress C/EBPδ is induced by the PERK pathway to contribute to the induction of the chemokines CXCL8 and CCL20, thus revealing a novel mechanism for ER stress-mediated modulation of cancer cells’ microenvironment.

## Results

### Endoplasmic reticulum stress induces C/EBPδ expression in cancer cell lines

**Fig. 1:**
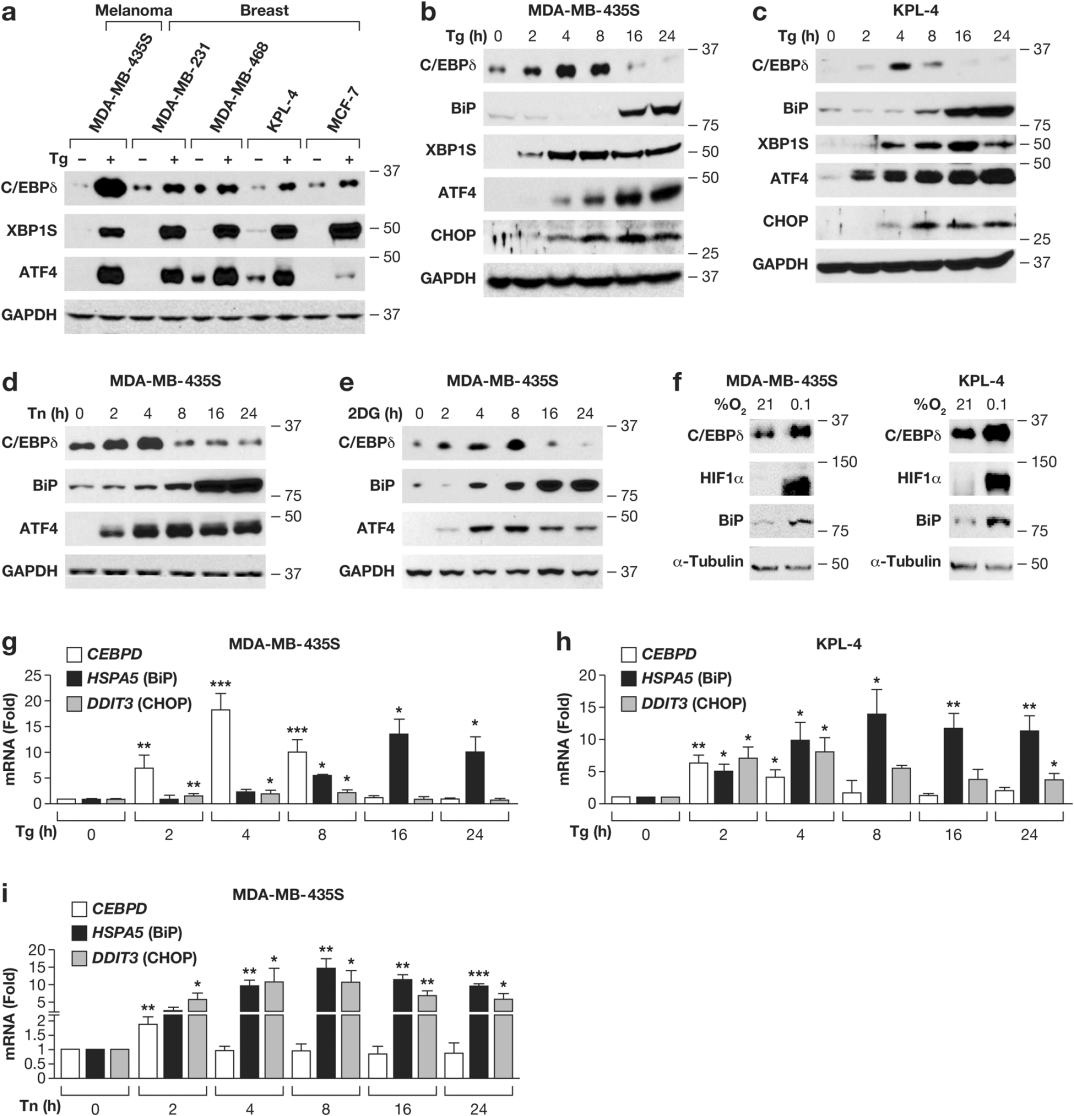
Endoplasmic reticulum stress modulates C/EBPδ expression in multiple cell lines. **a** Western blot analysis of the indicated proteins from cell lines representing melanoma and breast cancer treated with 100 nM of Thapsigargin (Tg) for 4 h. **b**–**e** Western blot analysis of **b** MDA-MB-435S or **c** KPL-4 cells treated with 100 nM Tg for the indicated times, **d** MDA-MB-435S cells treated with 10 µg/ml Tunicamycin (Tn) or **e** 10 mM 2-deoxy-glucose (2DG) for the indicated duration (0 h = vehicle treated for 24 h). **f** MDA-MB-435S and KPL-4 cells exposed to anoxia (0.1% O_2_) or ambient (21% O_2_) for 24 h. **a**–**f** labels on the right indicate molecular weight in kDa for this and subsequent Figures. **g**, **h** qRT-PCR analysis of mRNA levels as indicated in **g** MDA-MB-435S and **h** KPL-4 cells treated as in **b**. **i** qRT-PCR analysis of mRNA levels in MDA-MB-435S cells treated with 10 µg/ml Tn for the indicated times. Data are represented as mean±S.E.M, *n* = 3, *P < 0.05, **P < 0.01, ***P < 0.001.

To investigate C/EBPδ expression in response to ER stress, we first used thapsigargin, which induced C/EBPδ protein levels along with the UPR effectors XBP1S and ATF4 in multiple cancer cell lines representing melanoma and breast cancer (Fig. 1a). Kinetic analysis in MDA-MB-435S melanoma cells [19], showed that C/EBPδ induction peaked between 4–8 h, relatively concurrent with XBP1S, ATF4 and CHOP (Fig. 1b). The ER chaperone BiP/GRP78 was induced after the first wave of response, as shown previously [20]. Similar data were obtained in KPL-4 (Fig. 1c) and four additional breast cancer cell lines (MDA-MB-468, BT-549, MCF-7, MDA-MB-231) albeit with varying kinetics and relationships to XBP1S, ATF4, and/or CHOP expression (Fig. S1a–d). In contrast, cells with high basal levels of C/EBPδ [18, 21], such as the breast cancer cells SUM149 and SUM159, and mammary epithelial cell lines (MCF-10A and MCF-12A) rather reduced its expression in response to thapsigargin (Fig. S1e–h). To address C/EBPδ expression in response to other triggers of the UPR, we treated MDA-MB-435S cells with the N-glycosylation inhibitor tunicamycin, 2-deoxy-glucose, and anoxia [22, 23]. C/EBPδ was induced under all conditions (Fig. 1d–f), and similar results were obtained with breast cancer cell lines (Fig. S1i–l). Analysis of mRNA levels in KPL-4 and/or MDA-MB-435S cells showed that not only the protein but also *CEBPD* mRNA was induced by thapsigargin (Fig. 1g, h) as well as tunicamycin, albeit with lower amplitude and shorter kinetics (Fig. 1i). Taken together, these data show that *CEBPD* is an early ER stress response gene in multiple cancer cells with low basal level expression.

### JAK/STAT3 activation by PERK contributes to *CEBPD* gene expression

**Fig. 2:**
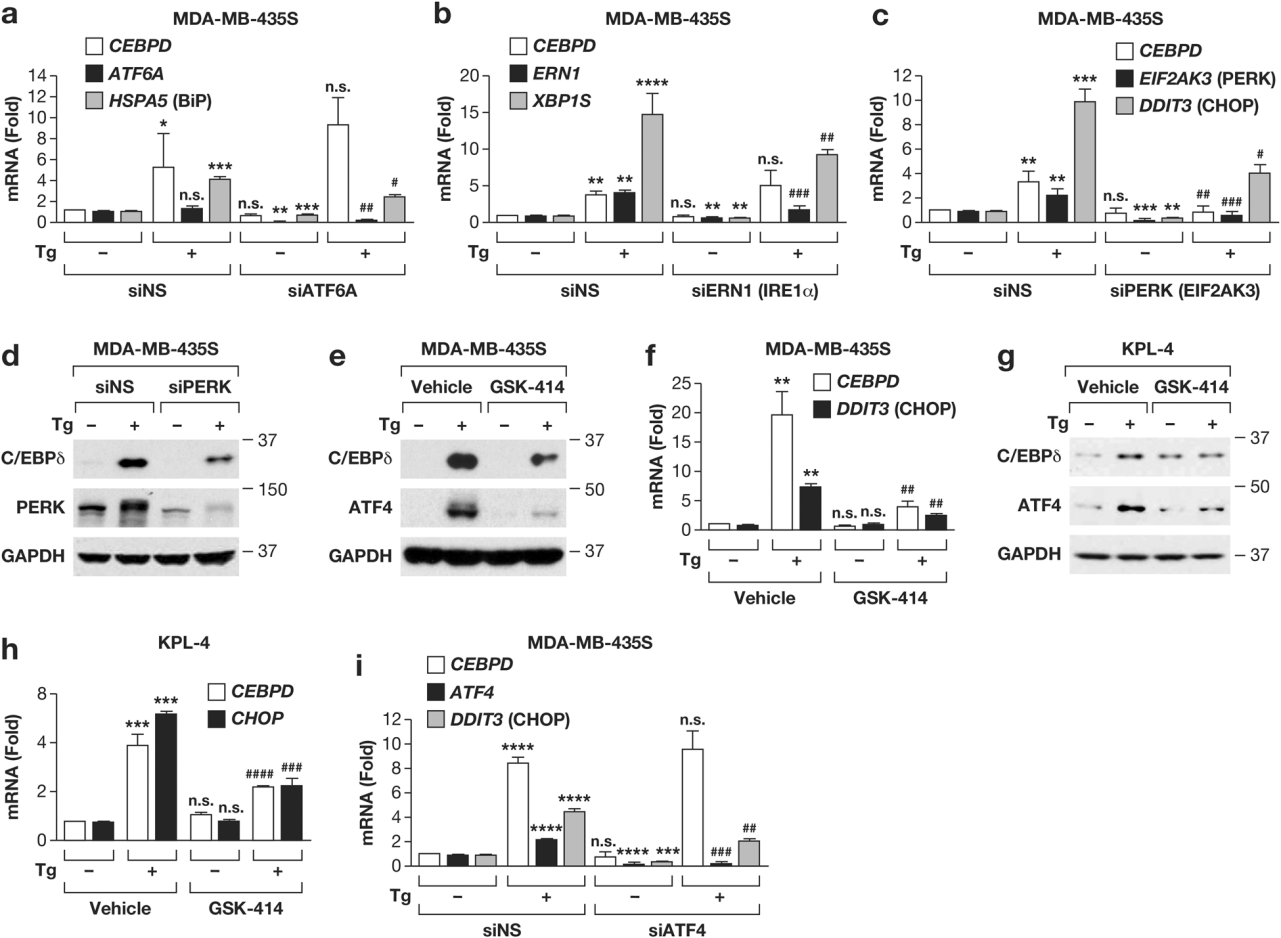
C/EBPδ is induced by the PERK pathway of the unfolded protein response in MDA-MB-435S and KPL-4 cells. **a**–**c** qRT-PCR analysis of the indicated mRNA levels in MDA-MB-435S cells transfected with control siRNA (siNS) or **a** siATF6A (ATF6α), **b** siERN1 (IRE1α), or **c** siPERK (targeting *EIF2AK3*), and treated for 6 h with 100 nM Tg (+) or DMSO (−). **d** Western blot analysis of MDA-MB-435S cells transfected with siRNA and treated with Tg (100 nM) for 3 h as indicated. **e**–**h** Western blot analysis (**e**, **g**) and qRT-PCR analysis (**f**, **h**) of MDA-MB-435S (**e**, **f**) and KPL-4 (**g**, **h**) cells treated with DMSO or Tg (100 nM) for 3 h and with or without pre-treatment with 1 µM GSK2606414 (GSK-414). **i** qRT-PCR analysis of the indicated mRNA levels in MDA-MB-435S cells transfected with siNS or siATF4 and treated with DMSO or Tg for 6 h. Quantitative data are represented as mean ± S.E.M, *n* = 3; *P < 0.05, **P < 0.01, ***P < 0.001, ****P < 0.0001 for comparisons to siNS or vehicle control *without Tg or #with Tg treatment, n.s., not significant.

To identify which arm of the UPR was responsible for *CEBPD* mRNA induction, we individually silenced the three ER transmembrane sensors in MDA-MB-435S cells. Knockdown of ATF6 or IRE1α (*ERN1)* resulted in downregulation of the canonical targets *HSPA5* (encoding BiP) and *XBP1S*, respectively, but did not perturb *CEBPD* induction by thapsigargin (Fig. 2a, b). In contrast, depletion of PERK (*EIF2AK3)* not only impaired induction of its canonical target *DDIT3* (CHOP) but also of *CEBPD* (Fig. 2c), which was confirmed at protein level (Fig. 2d). As alternative approach, the PERK kinase inhibitor GSK2606414 (GSK-414) [24] similarly reduced C/EBPδ and ATF4 induction by thapsigargin at the protein and mRNA level (Fig. 2e, f), and likewise in KPL-4 cells (Fig. 2g, h). Depletion of ATF4, a canonical mediator of PERK signaling, reduced induction of its target *DDIT3* [25] but not of *CEBPD* (Fig. 2i). In contrast to pharmacological inhibitor studies in MDA-MB-231 cells [26], we did not observe changes in the basal levels of *CEBPD* when inhibiting the three principal UPR effectors by RNAi approaches. These data show that, in response to thapsigargin, *CEBPD* mRNA expression is induced by the PERK pathway through ATF4-independent mechanisms.

To identify the mechanism of PERK induced *CEBPD* expression, we addressed the potential roles of other previously identified PERK substrates such as diacylglycerol (DAG), which activates PI3K-AKT-mTORC1 and RAS-RAF-MEK-ERK pathways, and the NRF2 transcription factor [27, 28]. Pharmacological inhibition of MEK (U0126), mTORC1 (rapamycin) and AKT (MK-2206) or NRF2 silencing, respectively, did not reduce C/EBPδ induction by Tg (Fig. S2a–d), which ruled out these pathways.

**Fig. 3:**
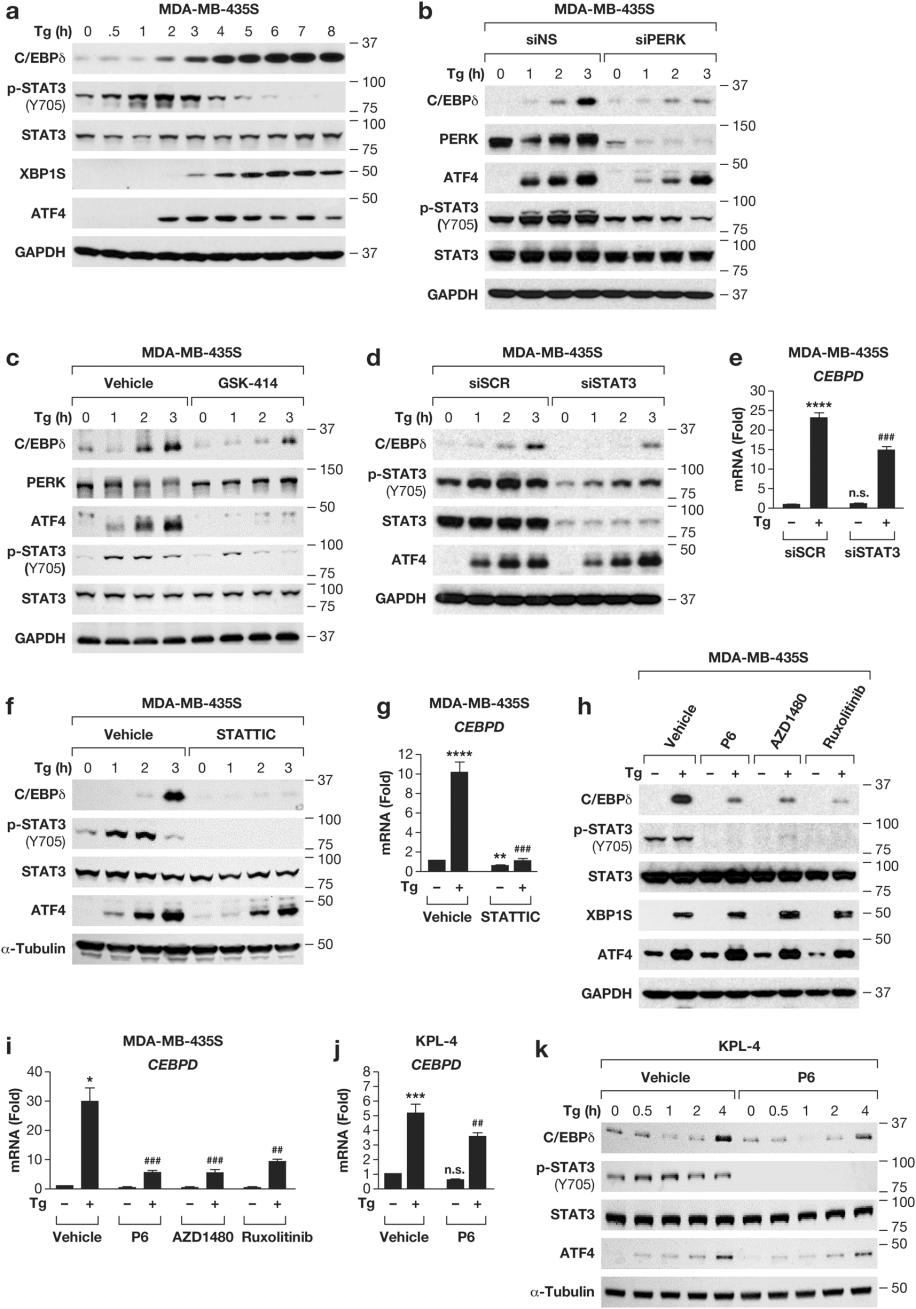
PERK-dependent JAK/STAT3 activation contributes to C/EBPδ induction. **a** Western blot analysis of the indicated proteins in MDA-MB-435S cells treated with 100 nM Tg for the indicated times (0 h = vehicle treated for 8 h). **b**, **c** Western blot analysis of MDA-MB-435S cells (**b**) transfected with siNS or siPERK, or **c** pre-treated with 1 µM GSK-414, and treated with Tg for the indicated times (0 h = vehicle treated for 3 h). **d** Western analysis of cells treated as in b after transfection with siSCR or siSTAT3. **e** qRT-PCR analysis of *CEBPD* mRNA in cells as in **d** treated for 3 h with Tg. **f** Western analysis of MDA-MB-435S cells as in **c** pre-treated with DMSO or STAT3 inhibitor STATTIC (20 µM). **g** qRT-PCR analysis of *CEBPD* mRNA in cells as in **f** treated for 3 h with Tg. **h**, **i** Western (**h**) and qRT-PCR (**i**) analysis of MDA-MB-435S cells treated with DMSO or Tg (100 nM) for 3 h and pre-treated with the JAK inhibitors Pyridone6 (P6, 1 µM), AZD1480 (1 µM) or Ruxolitinib (1 µM) as indicated. **j**, **k** qRT-PCR (**j**) and Western blot analysis (**k**) of KPL-4 cells treated with DMSO or Tg (100 nM) for 3 h (**j**) or as indicated (**k**) with or without pre-treatment with JAK inhibitor Pyridone6 (P6, 1 µM). Quantitative data are represented as mean±S.E.M, *n* = 3; *P < 0.05, **P < 0.01, ***P < 0.001, ****P < 0.0001 for comparisons to control siRNA or vehicle *without or #with Tg-treatment, n.s., not significant.

In astrocytes, PERK activation of STAT3 through direct interaction with and activation of the janus kinase JAK1 has been described [29]. The *CEBPD* promoter can be targeted by the STAT3 transcription factor which is activated by IL-6 [15]. Indeed, IL-6-mediated activation of STAT3 induced *CEBPD* expression also in MDA-MB-435S and KPL-4 cells (Fig. S2e, f). To address the potential role of a PERK-JAK-STAT3 pathway in *CEBPD* induction, we first monitored STAT3 activation in thapsigargin-treated MDA-MB-435S cells by its Y705 phosphorylation status [30], which increased transiently preceding the induction of C/EBPδ and the canonical UPR effectors (Figs. 3a and S2g). Inhibition of PERK by siRNA or GSK-414 attenuated C/EBPδ protein induction and STAT3 phosphorylation (Figs. 3b, c and S2h, i), while inhibition of the other pathways did not (Fig. S2g–i). Next, we used siRNA to deplete STAT3, which had no effect on thapsigargin-induced ATF4 expression but reduced C/EBPδ induction at the RNA and protein levels (Fig. 3d, e). As alternative approach, we used pharmacological inhibition of STAT3 by STATTIC [31], which completely abrogated *CEBPD* mRNA and protein induction (Fig. 3f, g). However, ATF4 induction was also attenuated, most likely due to STATTIC’s broad off-target effects [32]. Next, we assessed the role of JAKs with three inhibitors [33], all of which efficiently attenuated STAT3 phosphorylation and C/EBPδ mRNA and protein induction but did not significantly perturb induction of XBP1 and ATF4 protein (Fig. 3h, i). In KPL-4 cells, JAK inhibition also attenuated C/EBPδ mRNA and protein accumulation (Fig. 3j, k). Lastly, we asked whether glucose deprivation (GD) as a non-pharmacological trigger of ER stress, also activated this pathway. C/EBPδ protein expression, along with ATF4, was induced, but only after 8-24 h of GD while STAT3 phosphorylation was inhibited, and was not affected by GSK-414 (Fig. S2j, k). In contrast to its protein, *CEBPD* mRNA expression was downregulated (Fig. S2l). Thus, engagement of the JAK/STAT pathway by PERK may depend on the context and/or severity of ER stress, which is known to modulate the type of UPR [34]. Taken together, these results show that thapsigargin induces *CEBPD* expression through PERK and JAK kinases and in part by the STAT3 transcription factor.

### C/EBPδ regulates a subset of ER stress induced genes

**Fig. 4:**
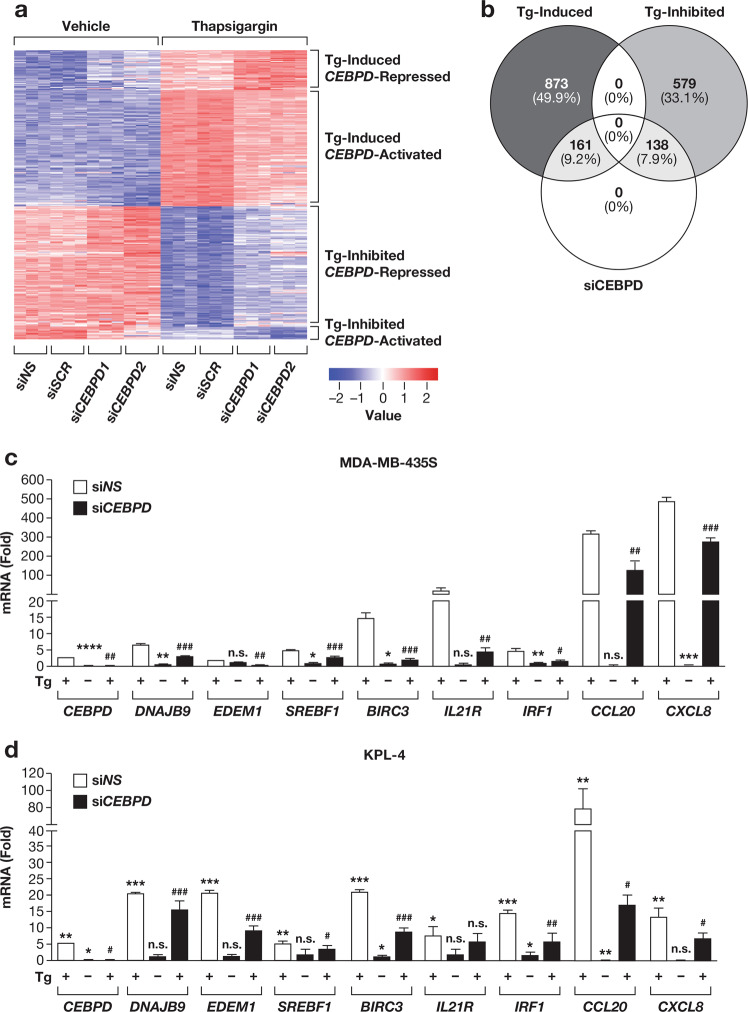
C/EBPδ mediates ER stress-induced changes in gene expression. **a** Heatmap of differentially expressed genes (DEGs) as determined by mRNA-Seq of MDA-MB-435S cells transfected with either of two independent controls (NS, SCR) or two independent *CEBPD*-targeting siRNAs (CEBPD1, CEBPD2) treated with vehicle or 100 nM Tg for 6 h. Only genes that were significantly altered by Tg-treatment in control siRNA transfected cells were included in this analysis, **b** Venn diagram illustrating the number of DEGS shown in **a**. **c** qRT-PCR analysis of select genes from **a** in siCEBPD1 transfected cells relative to DMSO-treated siNS control of cells treated as in **a**. **d** qRT-PCR analysis of the indicated genes in KPL-4 cells after transfection with siNS control or siCEBPD1 + 2 and treated with DMSO or Tg (100 nM) for 6 h, relative to DMSO treated control. Quantitative data are represented as mean±S.E.M, *n* = 3; *P < 0.05, **P < 0.01, ***P < 0.001, ****P < 0.0001 for comparisons to siNS treated with *DMSO or #Tg; n.s., not significant.

To determine the transcriptional program regulated by C/EBPδ during ER stress, we conducted an mRNA-Seq analysis. MDA-MB-435S cells were transfected with two independent *CEBPD* siRNAs or two independent controls followed by treatment with thapsigargin for 6 h. Among genes that were either induced or repressed by thapsigargin, 299 (20%) were significantly affected by *CEBPD* silencing (Fig. 4a, b). Because a reliable antibody for C/EBPδ ChIP-Seq was not available at the time, we mined prior ChIP datasets for binding to these genes’ promoters by any of the five C/EBP family proteins (C/EBPα, C/EBPβ, C/EBPγ, C/EBPδ, and C/EBPε) that share a common binding motif, which indicated the potential for C/EBPδ binding within the proximal gene promotors of 247 genes (Table S1). Ingenuity Pathway Analysis (QIAGEN Inc.) [35] revealed that the top Canonical Pathway represented by the differentially expressed genes (DEGs) was the “unfolded protein response” (*P* = 2.13E-09). “Cell death and survival” was the top altered Molecular and Cellular Function (*P* = 1.03E-18). PERK was among the most affected upstream regulators (EIF2AK3, *P* = 6.61E-13; Table S2), suggesting that C/EBPδ is one of the mediators of PERK signaling. For validation by qRT-PCR analysis of independent mRNA samples, we selected several of the Tg-induced/*CEBPD*-activated DEG’s relevant to ER homeostasis (Fig. 4c) and confirmed differential expression of genes that function in Endoplasmic Reticulum Associated Degradation (ERAD) such as *DNAJB9* and *EDEM1*, the stress-response transcriptional regulator of lipid homeostasis (*SREBF1)*, and the *BIRC3* gene encoding cellular inhibitor of apoptosis protein 2 (c-IAP2). Among secreted factors, *CXCL8* (encoding IL-8) and *CCL20* showed the highest fold-change due to si*CEBPD* and were validated along with *IL21R* and *IRF1* (Fig. 4c) as inflammatory signaling molecules. Similar results were obtained in the KPL-4 breast cancer cell line (Fig. 4d). Taken together, these data show that C/EBPδ, directly and/or indirectly mediates the expression of many genes in response to thapsigargin.

### C/EBPδ induces *CXCL8* and *CCL20* transcription

**Fig. 5:**
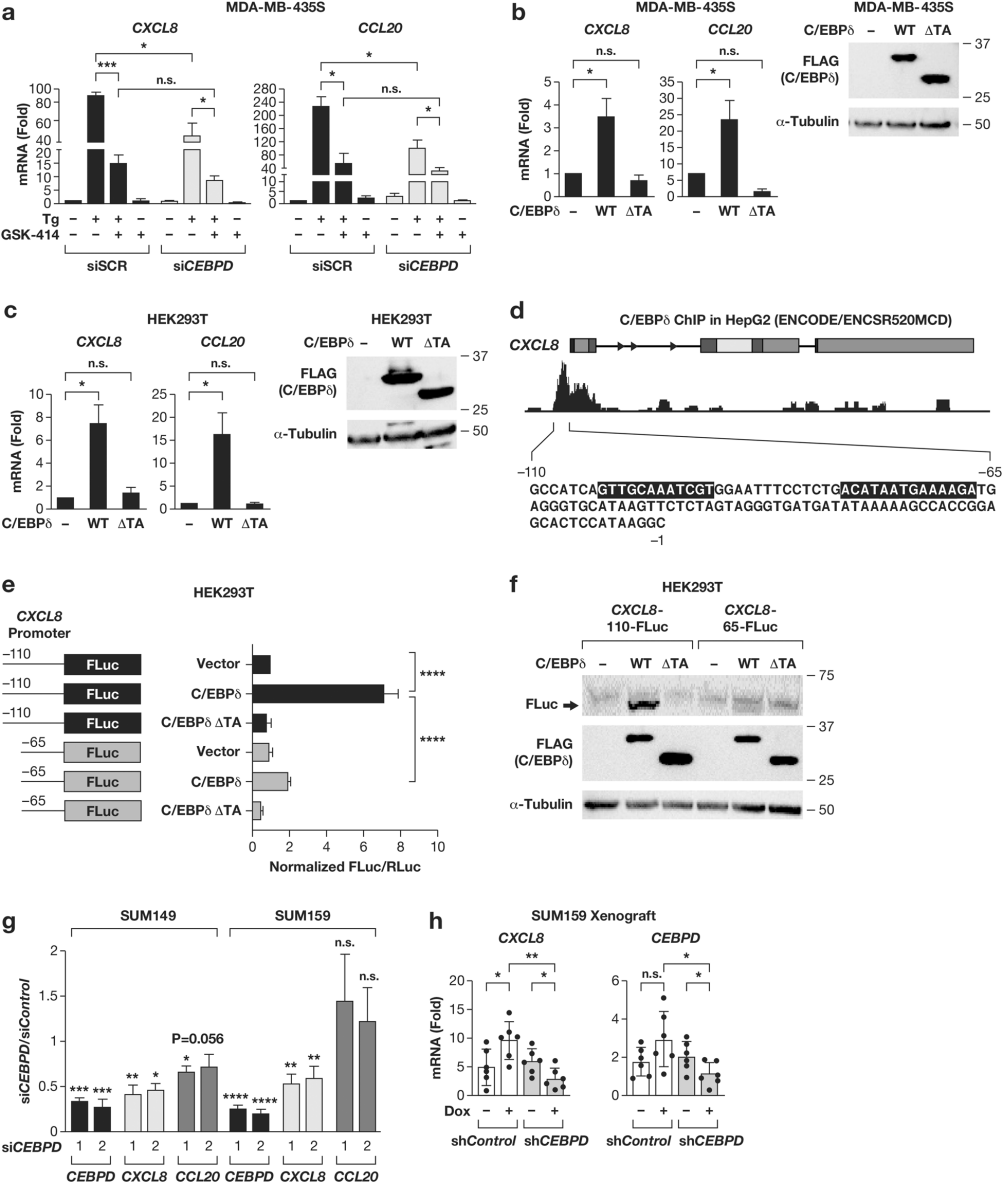
C/EBPδ is sufficient to induce *CXCL8* and *CCL20* transcription. **a** qRT-PCR analysis of *CXCL8* and *CCL20* mRNA levels in MDA-MB-435S cells transfected with siSCR or siCEBPD, treated with DMSO (−) or Tg (100 nM) for 6 h and with or without with GSK-414 (1 µM) pre-treatment as indicated. **b**, **c** qRT-PCR analysis of CXCL8 and CCL20 mRNA levels in MDA-MB-435S (**a**) and HEK293T (**b**) cells transfected with expressing constructs of full length (WT) or transactivation domain-truncated (ΔTA) C/EBPδ with FLAG-tag. Empty vector was used as control. Panels on the right show Western blots to demonstrate expression of ectopic C/EBPδ proteins. **d** Schematic of the *CXCL8* gene along with the C/EBPδ ChIP-Seq track from HepG2 cells as reported by the ENCODE database, and the sequence between positions −110 and −1 harboring a peak and C/EBP motifs (shaded) and indicating the position (−65) of the deletion mutation of the reporter construct shown in **e**. **e** Luciferase reporter assay in HEK293T cells co-transfected with C/EBPδ expression constructs as in **b** and Firefly luciferase (FLuc) reporter constructs containing the proximal CXCL8 promoter region from position −110 or −65 to −1. **f** Western blot analysis of representative extracts from **e** showing FLuc and C/EBPδ (anti-FLAG) protein expression. **g** qRT-PCR analysis of the mRNA levels of *CEBPD*, *CXCL8*, and *CCL20* in SUM149 and SUM159 cells 72 h after nucleofection with control or two independent siRNAs against *CEBPD*. **h** qRT-PCR analysis of *CXCL8* and *CEBPD* mRNA in SUM159 xenograft tumors with Dox-inducible shRNA and with and without Dox-treatment as described [18]. Quantitative data are mean±S.E.M; **a**–**c**, **g**, *n* = 3; **e**, *n* = 4; **h**, *n* = 6; *P < 0.05, **P < 0.01, ***P < 0.001, ****P < 0.0001; n.s., not significant.

Although *CEBPD* knockdown attenuated induction of pro-survival genes such as *BIRC3*, our pilot experiments did not suggest that C/EBPδ played a significant role in cell survival upon thapsigargin treatment. Thus, we focused our attention on the expression of the chemokines CXCL8 and CCL20. Inhibition of PERK in *CEBPD*-silenced cells further reduced the induction of *CXCL8* and *CCL20* mRNA expression by thapsigargin but did not completely inhibit their induction (Fig. 5a). These data indicate that *CXCL8* and *CCL20* are induced by C/EBPδ in cooperation with other PERK-activated factors [36, 37] as well as PERK-independent pathways. To further evaluate the role of C/EBPδ in the regulation of *CXCL8* and *CCL20*, we overexpressed C/EBPδ in MDA-MB-435S and HEK293T cells, which was sufficient to upregulate the endogenous mRNA levels of these chemokines, while a mutant without transactivation domain had no effect (Fig. 5b, c). C/EBPδ binding to the *CCL20* promoter has been reported [38]. C/EBPδ ChIP-Seq data from HepG2 cells indicated binding to a site in the proximal promoter of *CXCL8* (Fig. 5d). Transactivation of a *CXCL8* promoter reporter construct by C/EBPδ was significantly dependent on the presence of this region (Fig. 5e, f). These data show that C/EBPδ could induce gene expression in the absence of ER stress signals. To interrogate cells with high basal level of C/EBPδ, we silenced it in SUM149 and SUM159 cells and observed reduction in *CXCL8* in both lines and *CCL20* in SUM149 (Fig. 5g). Next, we analyzed xenograft tumors of SUM159 cells with doxycycline-inducible shRNA expression [18]. Tumors from doxycycline-treated control mice exhibited increased *CXCL8* expression, which may be due to increased ER stress [39] (Fig. 5h). However, when *CEBPD* was concomitantly silenced, *CXCL8* expression was reduced as well (Fig. 5h). *CCL20* expression was more variable and differences between groups not statistically significant. Taken together, these data show that C/EBPδ promotes expression of *CCL20* and/or *CXCL8* expression, most likely through direct binding to their endogenous promoters.

### Transient exposure to thapsigargin triggers secretion of CXCL8 and CCL20, paracrine modulation of immune cells, and cancer cell growth

**Fig. 6:**
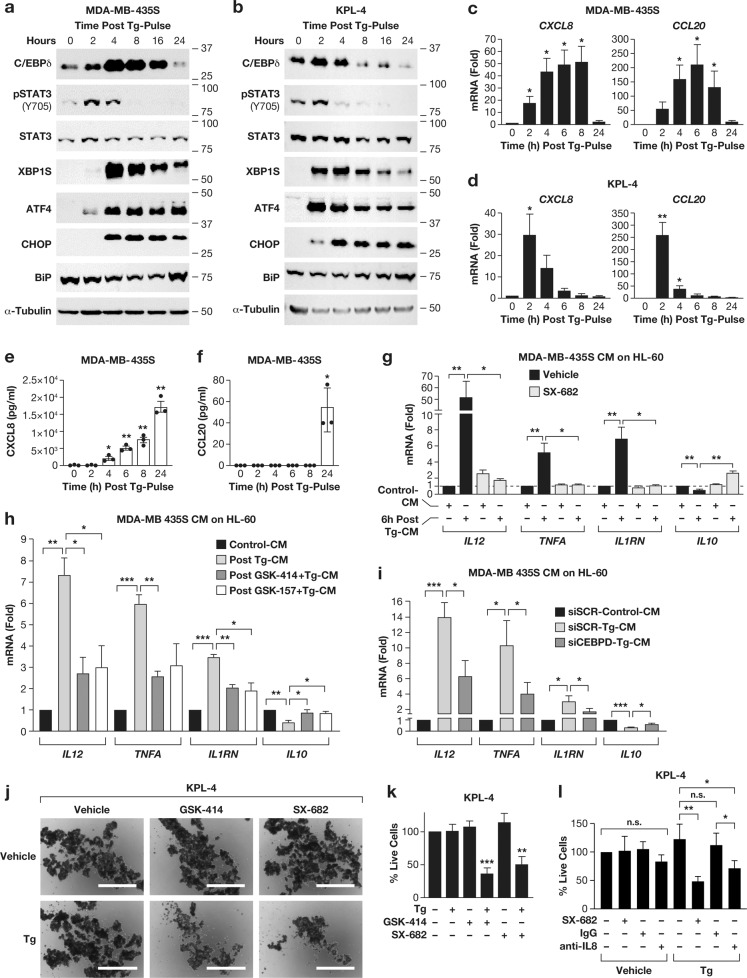
Transient exposure to Thapsigargin triggers long-term secretion of CXCL8 and CCL20 and modulation of immune and tumor cells. **a**, **b** Western blot analysis of the indicated proteins in (a) MDA-MB-435S and (b) KPL-4 cells treated with 100 nM Tg for 30 min followed by removal of Tg and collected at indicated times thereafter (0 h = pulse-treated with DMSO). **c**, **d** qRT-PCR analysis of *CXCL8* and *CCL20* mRNA levels in **c** MDA-MB-435S and **d** KPL-4 cells treated as panels **a** and **b**. **e**, **f** Enzyme-linked immunosorbent assay (*ELISA*) for **e** CXCL8 and **f** CCL20 in conditioned media from MDA-MB-435S cells treated as in **a**. **g** qRT-PCR analysis of *IL12, TNFA, IL1RN* and *IL10* mRNA levels in HL-60 cells treated for 24 h with 33% of 6 h-conditioned media from MDA-MB-435S cells pulse-treated with 100 nM Tg for 30 min. **h** qRT-PCR analysis of *IL12, TNFA, IL1RN* and *IL10* mRNA levels in HL-60 cells treated for 1 h with 33% of 6 h-conditioned media from MDA-MB-435S pulse-treated with 100 nM Tg for 30 min and PERK inhibitors GSK-414 or GSK2656157 (GSK-157) as indicated. **i** qRT-PCR analysis of gene expression in HL-60 cells as in **h** using CM from MDA-MB-435S cells transfected with siRNA and pulse-treated with Tg for 30 min as indicated. **j**, **k** KPL-4 cells by **j** light microscopy (Scale bar: 1 mm) and **k** live cell counts on day 4 of suspension culture that was preceded by 6 h of the indicated treatments with Tg (100 nM), GSK-414 (1 µM), and/or SX-682 (10 µM) in attachment culture (2D). **l** Live cell counts of KPL-4 cells on day 4 of suspension culture that was preceded by 6 h of the indicated treatments with Tg (100 nM), SX-682 (10 μM), IgG (20 μg/ml) or anti-IL8/CXCL8 (20 μg/ml) in attachment culture (2D). Quantitative data are represented as mean±S.E.M; *n* = 3, except **i** *n* = 4; *P < 0.05, **P < 0.01, ***P < 0.001, ****P < 0.0001.

The chemokines CXCL8 and CCL20 are modulators of immune cells as well as cancer cells [40, 41]. To assess the effect of ER stress-induced secreted factors, we treated cells with thapsigargin for 30 min followed by removal of the drug as described [42] and collection of conditioned media (CM). First, we confirmed that 30 min of thapsigargin exposure were sufficient to trigger lasting UPR pathway activation [43] in MDA-MB-435S and KPL-4 cells, indicated by increased expression of XBP1S, ATF4, CHOP, and C/EBPδ and STAT3 phosphorylation (Figs. 6a, b and S3). The mRNAs of *CXCL8* and *CCL20* were highly induced in both cell lines albeit with different kinetics (Fig. 6c, d). CXCL8 protein accumulated steadily in media from pulse-treated MDA-MB-435S cells, while CCL20 protein was only detectable after 24 h at low concentration (Fig. 6e, f). Prolonged activation of the UPR by short-term treatment is likely due to thapsigargin’s mechanism of irreversible inhibition of the SERCA protein [44].

Next, we incubated the human HL-60 promyelocytic cell line with CM from MDA-MB-435S cells that had been pulse-treated with thapsigargin (postTg-CM). First, we determined that postTg-CM treatment did not lead to measurable UPR activation in HL-60 cells, as the mRNA levels of *HSPA5*, *XBP1S* and *DDIT3* were not induced but did increase in response to thapsigargin (Fig. S4a, b). Next, we tested whether postTg-CM modulated inflammatory gene expression. Several genes encoding cytokines (*IL12, IL6, TNFA*) and the IL-1 receptor antagonist *IL1RA* were induced by postTg-CM, whereas expression of *IL10* was inhibited (Fig. 6g). Addition of SX-682, an inhibitor of the CXCL8 receptors CXCR1 and CXCR2 [45], blocked these responses (Fig. 6g). Similar results were obtained with postTg-CM from KPL-4 cells (Fig. S4c). We used SX-682 instead of antibody-mediated neutralization of CXCL8 because HL-60 cells express Fc receptors, which would trigger inflammatory gene expression [46, 47]. Therefore, we cannot rule out the potential role of other CXCR1/2 ligands [48]. However, the mRNA-Seq data did not indicate that any of them were induced by Tg. Nevertheless, two different PERK inhibitors or silencing of CEBPD in MDA-MB-435S cells significantly reduced the paracrine activity of post-TgCM on HL-60 gene expression (Fig. 6h, i). Taken together, our data demonstrate that PERK-C/EBPδ-dependent factors secreted from cancer cells after exposure to thapsigargin modulate myeloid cell gene expression through activation of CXCR1/2 receptors.

In breast cancer, CXCL8 promotes cancer cell stemness [49], which increases cells’ ability to grow as spheres in suspension culture (3D) [50]. To test the effect of UPR in this paradigm, KPL-4 cells were treated for 6 h with thapsigargin and/or GSK-414 or SX-682, followed by seeding in suspension without drugs. We found that KPL-4 cells proliferated robustly under these conditions, barring conclusions about selection of stem cells. However, both Tg+GSK-414 and Tg+SX-682 pre-treatments significantly reduced the number of live cells recovered after 4 d, while exposure to single agents had no effect (Fig. 6j, k). While *CXCR2* transcripts were not detectable in KPL-4 cells under any of these conditions, *CXCR1* transcript levels were low in 2D, modestly induced in 3D and more so in thapsigargin-treated cells (Fig. S5a). GSK-414, but not SX-682, completely abolished *CXCR1* induction (Fig. S5a). *CEBPD*-depletion showed that C/EBPδ also contributed to *CXCR1* expression in KPL-4 cells (Fig. S5b). Lastly, in this paradigm, antibody-mediated neutralization of CXCL8 during Tg-exposure reduced the number of live cells after 3D culture (Fig. 6l). Taken together, these data show that PERK and CXCR1 signaling poise thapsigargin-treated cells for survival and/or growth in suspension, which is mediated in part by CXCL8.

### PERK-C/EBPδ signaling leads to sustained CXCL8 and CCL20 expression

**Fig. 7:**
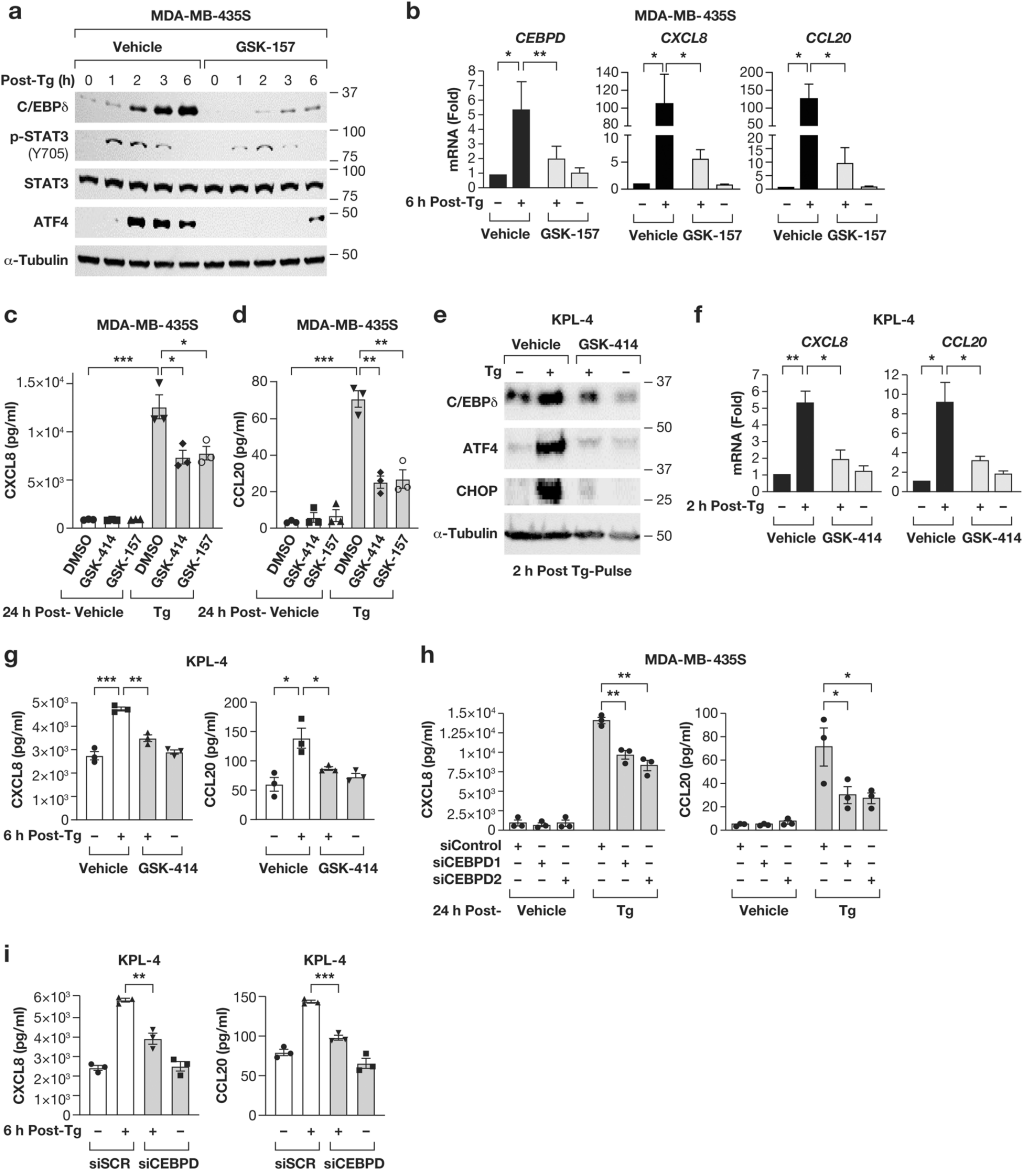
PERK and C/EBPδ contribute to chemokine expression following ER stress. **a** Western blot analysis of the indicated proteins from MDA-MB-435S cells collected at the indicated times after pulse-treatment with 100 nM Tg for 30 min with or without pre-treatment with GSK-157 (10 µM). **b** qRT-PCR analysis of *CEBPD*, *CXCL8* and *CCL20* mRNA level in MDA-MB-435S cells 6 h after pulse-treatment as in **a**. **c, d** *ELISA* for **c** CXCL8 and **d** CCL20 in 24 h conditioned media of MDA-MB-435S cells pulse-treated as in **a**. **e** Western blot analysis of indicated proteins from KPL-4 cells 2 h after pulse-treatment with 100 nM Tg with or without 30 min pre-treatment with 1 µM GSK-414. **f** qRT-PCR analysis of *CXCL8* and *CCL20* mRNA level from KPL-4 cells treated as in **e**. **g** ELISA of CXCL8 and CCL20 in 6 h conditioned media from KPL-4 cells treated as panel **e**. **h** ELISA of CXCL8 and CCL20 in 24 h conditioned media from MDA-MB-435S cells transfected with either control siRNA or two independent siRNAs against *CEBPD* and pulse treated with 100 nM Tg. **i** ELISA of CXCL8 and CCL20 in 6 h conditioned media from KPL4 cells transfected with either siSCR or si*CEBPD* and pulse treated with 100 nM Tg. Quantitative data are represented as mean±S.E.M, *n* = 3, *P < 0.05, **P < 0.01, ***P < 0.001.

Earlier, we showed that under chronic ER stress, PERK signaling induced pSTAT3 and C/EBPδ, which mediated *CXCL8* and *CCL20* induction. Therefore, we assessed whether this pathway also contributed to these chemokines’ expression after transient thapsigargin treatment. As seen with chronic treatment, PERK inhibition by GSK-157 during pulse treatment diminished STAT3 phosphorylation, C/EBPδ and ATF4 protein expression (Fig. 7a) and the induction of *CEBPD, CXCL8 and CCL20* mRNA (Fig. 7b) in MDA-MB-435S cells. Similar results were obtained with GSK-414 or PERK siRNA (Fig. S6a–d). Accordingly, PERK-silencing and both PERK inhibitors reduced the accumulation of CCL20 and/or CXCL8 in CM assessed at 6 h (Fig. S6e–g) and 24 h after pulse treatment (Fig. 7c, d). Similar results were obtained with KPL-4 cells (Fig. 7e–g). Lastly, silencing of *CEBPD* in MDA-MB-435S and KPL-4 cells also reduced the amounts of secreted CXCL8 and CCL20 (Fig. 7h, i). Taken together, these data confirm that the PERK-C/EBPδ pathway significantly contributes to chemokine expression after transient exposure to thapsigargin.

## Discussion

**Fig. 8:**
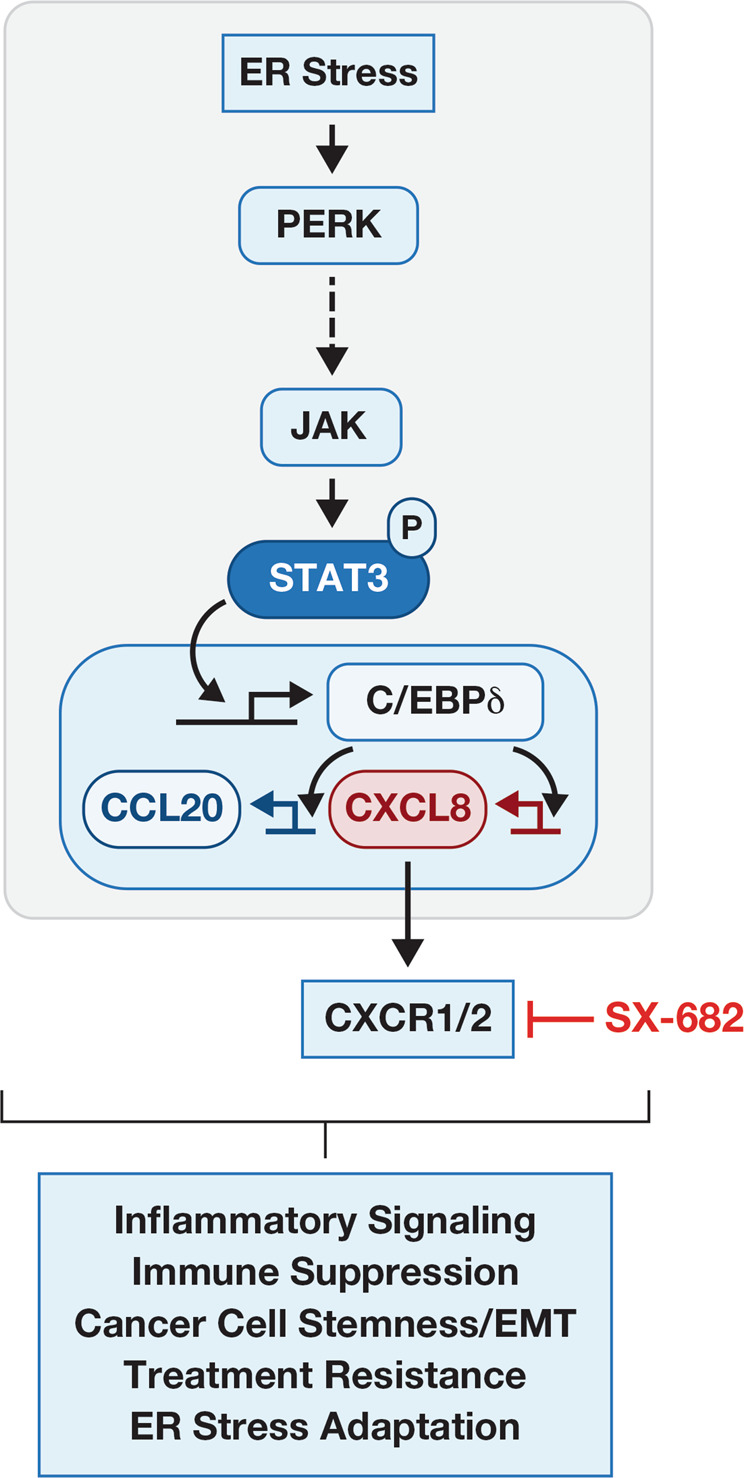
Model summarizing the pathway described in this study along with known effects of CCL20 and CXCL8 signaling in the context of cancer. Some of the relationships in this linear pathway may be the result of indirect mechanisms (indicated by dotted line) and/or also include the contribution of additional co-factors (not shown).

Autocrine and paracrine signaling by the tumor cell secretome are critical determinants of tumor biology by modulating the microenvironment and tumor cell phenotypes that contribute to tumor development and therapeutic outcomes. In this report, we describe a signaling pathway from PERK through C/EBPδ in cancer cells, which contributes to expression and secretion of CXCL8/IL-8 and CCL20 (Fig. 8), two chemokines that have well-documented tumor promoting effects through direct action on cancer cells as well as various immune cells [40, 48]. Activation of the STAT3 pathway may occur through direct interaction of PERK with JAK [29] or indirect mechanisms. In the context of immune cells, it is well established that the UPR triggers proinflammatory signaling [12, 51], including PERK-mediated induction of cytokine genes [29]. In cancer cells, IRE1 is reported to promote immunomodulatory cytokine expression [13] including CXCL8 [52]. PERK has been shown to induce *CXCL8* through NF-κB and ATF4 [36, 37]. Our study adds C/EBPδ to the arsenal of UPR mediators. In addition, we find that PERK-C/EBPδ can induce both *CXCL8* and *CXCR1*, thus issuing two-pronged activation of this pathway. Specifically, we show that cancer cells that had been exposed to thapsigargin, utilize this pathway for survival and growth in suspension.

C/EBPδ functions as a proximal ER stress responsive transcription factor that is transcriptionally induced at least in part through the JAK/STAT3 pathway. Studies in astrocytes revealed PERK-mediated activation of JAK-STAT3 signaling and induction of cytokines including CCL20 as a potential mechanism underlying neurological diseases [53]. Given that C/EBPδ can be expressed in astrocytes [54], we speculate that C/EBPδ may mediate some of PERK’s functions in these conditions. Here, we show that the PERK-JAK/STAT3 pathway can operate in certain cancer cell lines, adding to the molecular repertoire of ER stress-induced intra- and intercellular responses. The varying kinetics of C/EBPδ expression in different cancer cell lines may depend on the intrinsic variations of base line activation of the UPR and other pathways and/or balance of different arms of the UPR. For example, NF-κB is a candidate pathway that may also participate in ER stress-induced *CEBPD* transcription [15, 55]. On the other hand, the IRE1 pathway can inhibit C/EBPδ as seen by IRE1 deletion in mouse hepatocytes [56]. Thus, it may be interesting to assess if IRE1 is responsible for the transient nature of C/EBPδ induction in cancer cells experiencing ER stress, and/or for the reduction of C/EBPδ levels in cell lines with high basal expression.

Our transcriptomic study showed that C/EBPδ contributed to the regulation of about 20% of the thapsigargin-modulated transcriptome in MDA-MB-435S cells. The spectrum of direct and indirect C/EBPδ targets will certainly depend on the trigger for ER stress and cell type. In addition, it remains to be determined to what extent the potential interaction of C/EBPδ with other UPR effectors [26, 57], including ATF4 [37], may be involved in the regulation of these genes. Previous reports had implicated C/EBPδ in both activation and inhibition of *CCL20* and *CXCL8* expression in immune cells or brain cells [26, 58]. We show that C/EBPδ supports expression of these chemokines in cancer cells under acute ER stress as mediator of PERK activation. CXCL8 activation by C/EBPδ is also in line with its role in promoting cancer stem cells and tumor progression [18, 59]. We focused functional studies on the CXCL8-CXCR1/2 axis because CCL20 was expressed at significantly lower levels. However, within the tumor microenvironment, such levels of CCL20 expression may be biologically significant as an autocrine factor and/or modulator of infiltrating immune cells.

Because of the janus-faced nature of PERK signaling in cancer and the toxicity of PERK inhibitors in vivo, direct inhibition of PERK is currently not attainable in the clinic [27, 60, 61]. However, inhibition of IL-8 signaling is being investigated in the clinical setting. SX-682 is in clinical trials for melanoma (NCT03161431) and other CXCR1/2 inhibitors are in trials for combination therapies in breast cancer [62–64]. Mice lack the gene encoding CXCL8/IL-8 and the CXCR1 receptor may not be functionally identical to human [65]. Therefore, pre-clinical studies on the role of this pathway in the tumor-microenvironment interactions are not readily feasible. Nonetheless, our findings suggest that CXCR1/2 inhibitors could be suitable for combination therapy with ER stress inducing agents. Many chemotherapeutics disrupt cancer cell proteostasis and thereby cause ER stress [22]. On the other hand, the cancer cell secretome, and prominently IL-8, have been shown to contribute to treatment resistance [66, 67]. Our study contributes a molecular pathway from PERK to CXCL8/IL-8 as a plausible mechanism for emergence of immune suppression and resistance in response treatment.

## Materials and methods

### Reagents and antibodies

Thapsigargin (#586005) and Tunicamycin (#654380) were from Calbiochem, Burlington, MA, USA; PERK inhibitor GSK2606414 (GSK-414, #5107) from Tocris Minneapolis, MN, USA; JAK inhibitors Pyridone 6 (#420099), AZD1480 (#SML1505) and 2-deoxy-glucose (2DG, #D8375) from Millipore Sigma, St. Louis, MO, USA; IL-6 (#PHC0064) from Gibco-Life Technologies Corporation, Carlsbad, CA, USA; MK-2206 (#S1078) from Selleck Chemicals, USA); SX-682 (#HY-119339) and GSK-157 (GSK2656157, #HY-13820) from MedChemExpress, Monmouth Junction, NJ, USA; Ruxolitinib (#DC4230) from DC Chemicals, Shanghai, China; U0126 (#V112A) from Promega, Madison, WI, USA; rapamycin (#R-5000) from LC Laboratories, Woburn, MA, USA. 2DG was diluted in water, and all other agents in DMSO. Antibodies were obtained from Cell Signaling, Danvers, MA, USA (PERK, #3192; phospho-STAT3, #9145; STAT3, #4904; Phospho-Erk1/2, #9106; phospho-Akt, #4060; Akt, #4691; phospho-p70 S6 Kinase, #9234; p70 S6 Kinase, #9202; anti-mouse HRP, #7076; Anti-rabbit-HRP, #7074); Novus, Centennial, CO, USA (HIF-1α, #NB100-449); Santa Cruz Biotechnology, Dallas, Texas, USA (ATF4, sc-390063; CHOP, sc-4066; GAPDH, sc-47724; C/EBPδ, sc-135733); BD Pharmingen, San Jose, CA, USA (BiP, 610978); Biolegend, San Diego, CA, USA (XBP1S, 619502); Rockland, Limerick, PA, USA (Tubulin, 600-401-880); Promega (Fluc, G7451); Developmental Studies Hybridoma Bank, Iowa City, IA, USA (alpha-tubulin, #12G10, deposited by Frankel, J. / Nelsen, E.M); Millipore Sigma (anti-FLAG, #F1804), and R&D systems, Flanders, NJ, USA (anti-hCXCL8/IL-8, #AB-208-NA, IgG, #AB-108-C).

### Cell lines and culture

MDA-MB-435S, MDA-MB-231, MDA-MB-468, BT-549, HEK293T, HL-60 and MCF-7 originated from ATCC, Manassas, VA, USA. SUM149 and SUM159 cells originated from Asterand Bioscience. KPL-4 cells were established by Dr. Junichi Kurebayashi (Kawasiki Medical School) and kindly provided by Dr. Naoto Ueno (MDACC). All cell lines were routinely tested for mycoplasma and were authenticated bi-annually by Genetica (Labcorp, Burlington, NC, USA). Cells were cultured in DMEM supplemented with 10% fetal bovine serum (FBS) and 1% Penicillin-Streptomycin, except KPL-4 (DMEM/F12), HL-60 (RPMI), and SUM149 (Ham’s F-12 with 5 μg/ml hydrocortisone and 1 μg/ml Insulin). For MCF-7 and HEK293T, the medium was supplemented with 1 mM sodium pyruvate. MCF10A and MCF12A were cultured in DMEM + F12 GlutaMax supplemented with 5% FBS, 10 mg/ml Insulin (Millipore Sigma #I0516), 100 ng/ml Cholera toxin (Millipore Sigma #227036), 0.5 mg/ml Hydrocortisone (Millipore Sigma #H4001), 20 ng/ml EGF (Invitrogen, Carlsbad, CA, USA, #13247-051) and 1 mM CaCl_2_. Pre-treatments with inhibitors were for 30 min except SX-682 (15 min). Vehicle was used as control in all experiments. For anoxic treatment, cells were cultured 24 h in an anaerobic chamber with 0.1% O_2_. For glucose deprivation (GD), complete medium was removed, cells were washed twice with PBS followed by culture in no-glucose-DMEM (Thermo Scientific #11966025) supplemented with 10% dialyzed FBS (Thermo Scientific # A3382001) and 1% Penicillin-Streptomycin.

For suspension culture, 100,000 KPL-4 cells were first seeded in 6 well plates and the next day treated with GSK-414 (1 µM) and/or SX-682 (10 µM), and/or anti-hCXCL8/IL8 (20 µg/ml) and/or anti-IgG for 30 min prior to exposure to thapsigargin (100 nM) for 6 h. Cells were washed, trypsinized, and 2500 cells were seeded in 24 well ultra-low attachment plates (CORNING, St. Louis, MO, USA, #3473) in MammoCult medium supplemented with heparin and hydrocortisone (STEMCELL Technologies Seattle, WA, USA, #05620, #07925, #07980). After 4 days of suspension culture, cells were collected and centrifuged at 1000 rpm for 1 min and washed once with PBS. Cells were suspended in 0.5 ml culture medium and live cells were counted based on trypan blue dye-exclusion method.

#### Pulse treatment and condition media preparation

At about 70% confluence, cells were incubated with 100 nM thapsigargin (Tg) or 0.1% DMSO for 30 min followed by removal of the media. As applicable, inhibitors were added 30 min before Tg. Cells were washed twice with warm PBS, and then incubated with fresh medium for the indicated time points. Conditioned medium (CM) was collected, centrifuged for 10 min at 300 g to remove any cells. Target cells were incubated with a final concentration of 33% (v/v) CM for the indicated times. Time points for subsequent analyses were chosen based on the expression kinetics of the genes/proteins of interest in the respective cell lines and treatment paradigms.

### Transient transfections and RNAi

Cells were transfected with siRNA by nucleofection using the Amaxa Cell-line Nucleofector Kit V (Cat# VCA-1003; Lonza AG). Controls were Scrambled siRNA (SCR) (#D-001960-01-05, Dharmacon, Lafayette, CO, USA) or EGFP oligonucleotides (NS; 5′-CAAGCTGACCCTGAAGTTC-3′) [68]. Transfected cells were re-seeded 48 h post-transfection and allowed to attach overnight before starting treatments.

siRNA sequences for *CEBPD* were as follows:

CEBPD siRNA-1-sense 5′-rUrCrGrCrCrGrArCrCrUrCrUrUrCrArArCrArGTT-3′

CEBPD siRNA-1-antisense 5′-rCrUrGrUrUrGrArArGrArGrGrUrCrGrGrCrGrATT-3′

CEBPD siRNA2-sense 5′-rCrCrArCrUrArArArCrUrGrCrGrArGrArGrArATT-3′

CEBPD siRNA2-antisense 5′-rCrUrGrUrUrGrArArGrArGrGrUrCrGrGrCrGrATT-3′

siRNA pools for *ATF6A*/ATF6α (sc-37699), *ERN1*/IRE1α (sc-40705), *EIF2AK3*/PERK (sc-36213), *NRF2* (sc-37030) and *STAT3* (sc-29493) were purchased from Santa Cruz Biotechnology.

### RNA isolation and qRT-PCR

RNA was isolated using GeneJET RNA purification kit (Thermo Fisher Waltham, MA, USA), and cDNA was synthesized with Superscript reverse transcriptase III (RT) according to the manufacturer’s instructions (Invitrogen, CA). RNA samples of SUM159 cell xenograft tumors with Dox-inducible shRNA were as described [18]. qRT-PCR was performed with appropriate primer sets as described in Table S3 using Fast SYBR Green master mix (Applied Biosystems, Foster City, CA, USA) on the 7500 Fast or QuantStudio 5 Real Time PCR instrument (Applied Biosystems). Fold changes were analyzed using ddCt method and normalized to GAPDH or RPLP0. All reactions were performed in triplicates and the data are represented as mean ± S.E.M of at least three independent experiments as indicated.

### mRNA-Seq analysis and public data source

MDA-MB-435S cells were transfected with the indicated siRNA, 48 h later cells were treated with DMSO or 100 nM thapsigargin for 6 h and RNA was isolated using the RNAeasy kit (Qiagen, Germantown, MD, USA). mRNA-Seq analysis was carried out on samples from three biological replicates. Library preparation was done by following standard IlluminaTruSeq protocol FC-122-1001 with barcoding. The 24 samples were pooled and sequenced on two lanes of HiSeq2500 with Illumina TruSeq V4 chemistry, which yielded ~40 million strand-specific paired-end 125 bp reads with > 91% bases having call quality of Q30 or above per sample. Both reads of the samples were trimmed for adapters and low-quality bases using Trimmomatic software (version 0.30). The trimmed fastq data were aligned to human genome hg19 with STAR (version 2.4.2a), which used GENCODE gtf file version 19 (Ensembl 74). STAR software also generated the strand-specific gene read counts. About 80% of ~40 million reads per sample were mapped to the human genome uniquely for a total of ~87% mapping rate. The read counts for each gene from STAR were normalized with R Package limma (version 3.26.8). The differentially expressed genes between treated and control groups were analyzed with linear mixture models in R package lme4 (version 1.1-12). The heatmap display of manually curated genes with FDR < 0.05 between treated and control groups was generated with R packages gplots (version 3.0.1). The mRNA-Seq data are available at the NCBI Gene Expression Omnibus under accession number GSE131048. The Venn diagram was generated with the public software Venny (Oliveros, J.C. (2007-2015) at https://bioinfogp.cnb.csic.es/tools/venny/index.html.

### In silico ChIP analysis

Transcription factor binding regions (TFBRs) for the C/EBP family on the human genome (hg38) were obtained from meta-clusters reported in the Gene Transcription Regulation Database (https://gtrd.biouml.org/) [69]. These TFBRs were intersected with promoter regions of protein-coding genes, which were defined as 2 kb upstream and 500 bp downstream from the transcription-start-sites (TSS). Human gene annotations release 30 for hg38 from Genecode (https://www.gencodegenes.org/human/release_30.html) was used to determine TSSs. This analysis yielded a total of 15,591 genes, with an overlap of 247 genes in common with the mRNA-Seq DEGs as defined in Fig. 4a. ENCODE data for C/EBPδ binding to the *CXCL8* promoter were generated with an anti-Flag tag antibody against endogenously tagged *CEBPD* (ENCSR520MCD at www.encodeproject.org).

### Western blotting

All Western data were generated from whole cell lysates. Cells were scraped into medium and pelleted by centrifugation along with the floating population, washed once with cold PBS, lysed with 2x Laemmli sample buffer (Bio-Rad Laboratories, Hercules CA, USA) and heated at 100 °C for 5 min. Proteins were quantified using the Pierce 660 Protein Assay kit (Thermo Fisher Scientific, #22662) and loaded onto 4-20% SDS polyacrylamide gels (Invitrogen). After electrophoresis, proteins were transferred to nitrocellulose membrane and blocked with 5% non-fat dry milk in Tris Buffered Saline (TBS). Immunodetection was performed with the indicated primary antibodies for overnight incubation at 4 °C. Following washing and incubation with the appropriate HRP-conjugated secondary antibodies, signals were visualized by Supersignal West Dura Extended Duration substrate (#34076, Pierce, Rockford, IL, USA) or ProSignal Femto ECL reagent (#20-302, Genesee Scientific, El Cajon, CA, USA). All western analysis data are representative of at least three independent biological replications. Quantifications were done by ImageJ (https://imagej.nih.gov/ij/). After background subtraction, pSTAT3 data were normalized to STAT3 total protein and graphed as fold change with respect to untreated control from at least three independent experiments.

### Enzyme-linked immunosorbent assay

ELISA assays with conditioned media were performed using ELISA assay kits from R&D Systems (CXCL8 #D8000C, CCL20 #DM3A00) as per manufacturer’s instruction. Optical density of the products was measured with a SpectraMax iD3 instrument at 450 nm with a correction wavelength at 570 nm. Concentrations were estimated using standard curves generated with provided purified protein standards.

### Cloning of reporter and expression constructs and luciferase reporter assay

To mutate the transactivation domain of full-length Flag-tagged C/EBPδ [70], residues 45-85 were deleted by site directed mutagenesis (Agilent, Santa Clara, CA, USA, #210518) using the following primers: 5’GGGGCCCTAGGCGAGTTCAACAGCAATCACA-3’ and 5’-TGTGATTGCTGTTGAACTCGCCTAGGGCCCC-3’. The CXCL8 promoter regions were amplified from human genomic DNA with the following primers: IL8p-Fw1-XhoI (for −110: 5’-ACTCGAGCCATCAGTTGCAAATCGTG-3’) or IL8p-Fw2-XhoI (for −65: 5’- ACTCGAGGGTGCATAAGTTCTCTAGT-3’) and IL8p-Rev-HindIII (5’-TAAGCTTGCCTTATGGAGTGCTCCGGT-3’) and cloned into pGL3-Basic vector (Promega, E1751). HEK293T cells were co-transfected with CXCL8 promoter reporter constructs along with C/EBPδ expression constructs [70]. Renilla luciferase expression plasmid, pRL-CMV (Promega E2261) was used as an internal transfection control. Dual luciferase activity was assessed 48 h after transfection from equal amounts of total protein using Dual Luciferase assay kit (Promega, E1910) according to the manufacturer’s protocol. Luminescence was measured using a SpectraMax iD3 instrument.

### Statistical analysis

Unless stated otherwise, quantitative data were analyzed by the two-tailed unequal variance *t*-test and are shown as mean±S.E.M of at least three independent biological replicates using GraphPad Prism, version 8, La Jolla California USA. *p*-values *P < 0.05, **P < 0.01, ***P < 0.001, ****P < 0.0001 are designated as statistically significant and *P* ≥ 0.05 are designated as not significant (n.s.).

## Supplementary information

Supplementary Information

Table S1

Table S2

## Data Availability

The mRNA-Seq data are available at the NCBI Gene Expression Omnibus under accession number GSE131048.
