# Supplementary material for: PERK signaling through C/EBPδ contributes to ER stress-induced expression of immunomodulatory and tumor promoting chemokines by cancer cells
Source: Cell Death Dis. 2021 Nov 1;12(11):1038. doi: 10.1038/s41419-021-04318-y (PMC8560861; doi:10.1038/s41419-021-04318-y)
Supplement: Supplementary file 1 — Supplementary Information [file 41419_2021_4318_MOESM1_ESM.pdf]

PERK signaling through C/EBPδ contributes to ER stress-induced expression of immunomodulatory and tumor promoting chemokines by cancer cells

Namratha Sheshadri, Dipak K. Poria, Shikha Sharan, Ying Hu, Chunhua Yan, Vishal N. Koparde, Kuppusamy Balamurugan, and Esta Sterneck

Supplementary Information

In this file:

- **Supplementary Figure Legends**
- **Supplementary Figures S1-S6**
- **Table S3:** Primers used for q-PCR

Excel files, separate:

- **Table S1:** 247 common elements in Genome and 300 mRNA genes of Heatmap Figure 4
- **Table S2:** Ingenuity Pathway Analysis of mRNA-Seq data

PERK signaling through C/EBPδ contributes to ER stress-induced expression of immunomodulatory and tumor promoting chemokines by cancer cells

Namratha Sheshadri, Dipak K. Poria, Shikha Sharan, Ying Hu, Chunhua Yan, Vishal N. Koparde, Kuppusamy Balamurugan, and Esta Sterneck

Supplementary Figure Legends

Figure S1: C/EBPδ expression in response to ER stress inducing agents in cancer cell lines and non-transformed mammary epithelial cells.
a-l Western analysis of the indicated proteins' expression in response to 100 nM thapsigargin (Tg) at the indicated times in the human breast cancer cell lines (a) MDA-MB-468, (b) BT-549, (c) MCF-7, (d) MDA-MB-231, (e) SUM159, and (f) SUM149, and the breast epithelial cell lines (g) MCF-12A and (h) MCF10A; or (i) after treatment with Tunicamycin (Tn, 10μg/ml) in MCF-7 cells; (j) in response to anoxia 0.1% O_2 for 24 h in MDA-MB-468 cells; and in response to 2-deoxy-glucose (2DG, 10mM) for the indicated times in (k) MDA-MB-468 and (l) KPL-4 cells.

Figure S2: Analysis of the role of PERK signaling and STAT3 in C/EBPδ expression.
a-c Western blot analysis of indicated proteins from MDA-MB-435S cells treated with Tg for the indicated times either pre-treated with (a) 10 μM MEK inhibitor (U0126) or 50 nM rapamycin, or (b) 1 μM AKT inhibitor (MK-2206), or (c) transfected with the indicated siRNA (c). **d** qRT-PCR analysis of *NRF2* mRNA levels in MDA-MB-435S cells treated as in panel (c). **e** Western blot analysis of the indicated proteins in KPL-4 (left) and MDA-MB-435S (right) cells transfected with siSCR or siSTAT3 and two days later treated with IL-6 (100 ng/ml) for 16 h. **f** qRT-PCR analysis of *CEBPD* mRNA levels in KPL-4 (left) and MDA-MB-435S (right) treated as in panel (e). **g-i** Densitometric quantitation of phospho-STAT3 normalized to total STAT3 from biological replicates corresponding to the experiment in (g) Figure 3a, (h) Figure 3b and (i) Figure 3c. **j** Western blot analysis of indicated proteins from MDA-MB-435S cells glucose-deprived (GD) for the indicated time points (0h = complete media for 24h). **k** Western blot analysis of MDA-MB-435S cells glucose-deprived for 24 h with or without GSK-414 (1 μM) pre-treatment. **l** qRT-PCR analysis of *CEBPD* mRNA levels in MDA-MB-435S cells glucose-deprived for indicated times. Quantitative data are represented as mean±S.E.M; n=3, except (i) n=4; $^*P<0.05$, $^{**}P<0.01$, $^{***}P<0.001$, n.s., not significant.

Figure S3: Quantitation of STAT3 phosphorylation in response to thapsigargin.
Densitometric quantitation of phospho-STAT3 normalized to total STAT3 from three biological replicates corresponding to the experiments in Figure 6a (MDA-MB-435S) and Figure 6b (KPL-4); mean±S.E.M, n=3; $^*P<0.05$, $^{**}P<0.01$, $^{***}P<0.001$, n.s., not significant.

Figure S4: Analysis of gene expression in HL-60 cells in response to conditioned media.
a qRT-PCR analysis of *HSPA5*, *XBP1S* and *DDIT3* mRNA levels in HL-60 cells treated for 24 h with 33% of 6 h-conditioned media from MDA-MB-435S cells pulse-treated with 100 nM Tg for 30 min. **b** qRT-PCR analysis as in panel (a) of HL-60 cells treated with 100 nM Tg for 24 h. **c** qRT-PCR analysis of the indicated mRNA levels in HL-60 cells treated for 24 h with 33% of media, conditioned for 6 h by KPL-4 cells after 30 min pulse treatment with Tg (100 nM), with or without pretreatment with SX-682 (10 μM). Data are represented as mean±S.E.M, n=3; *P<0.05, **P<0.01, ***P<0.001.

Figure S5: Analysis of CXCR1 gene expression in KPL-4 cells.
a qRT-PCR analysis of *CXCR1* mRNA in KPL-4 cells in 2D or treated as in Figure 6j, compared to untreated cells in 3D culture or as indicated. **b** qRT-PCR analysis of *CEBPD* and *CXCR1* mRNA levels from KPL-4 cells transfected with siSCR and siCEBPD oligos for 18 h and treated with 100 nM Tg for 6 h in attachment culture (2D). Quantitative data are represented as mean±S.E.M, n=3; ; *P<0.05, **P<0.01, ***P<0.001, ****P<0.0001, n.s., not significant.

Figure S6: Analysis of the role of PERK in CXCL8 and CCL20 expression.
a Western blot analysis of the indicated proteins from MDA-MB-435S cells 6 h after pulse-treatment with Tg (100 nM) for 30 min and with or without 30 min GSK-414 pre-treatment. **b** qRT-PCR analysis of *CXCL8* and *CCL20* mRNA level in MDA-MB-435S cells treated as in panel (a). **c** Western blot analysis of MDA-MB-435S cells 2 days after transfection with siSCR or siPERK and 6 h after pulse-treatment with Tg (100 nM) for 30 min. **d** qRT-PCR analysis of *CXCL8* and *CCL20* mRNA level in MDA-MB-435S cells treated as in panel (c). **e** ELISA for CXCL8 in 6 h conditioned media of MDA-MB-435S cells treated as in panel (c). **f-g** ELISA for CXCL8 in 6 h conditioned media of MDA-MB-435S pulse-treatment with 100 nM Tg for 6h, with or without 30 min GSK-414 (f) or GSK-157 (g) pre-treatment. Quantitative data from at least three biological replicates are represented as mean±S.E.M; n=3, except (b) n=4; *P<0.05 , **P<0.01, ***P<0.001.

Supplemental Figure 1

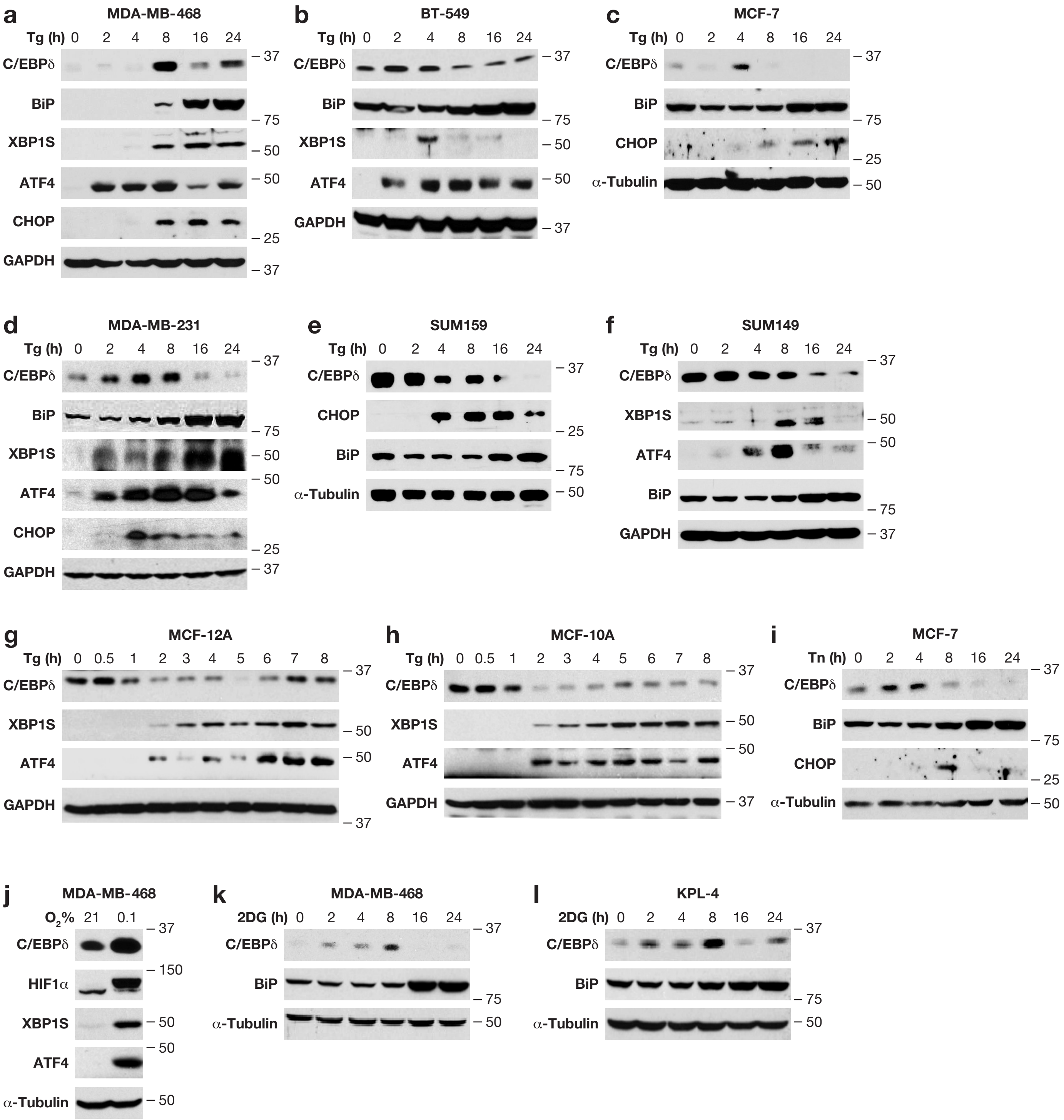

Supplemental Figure 2

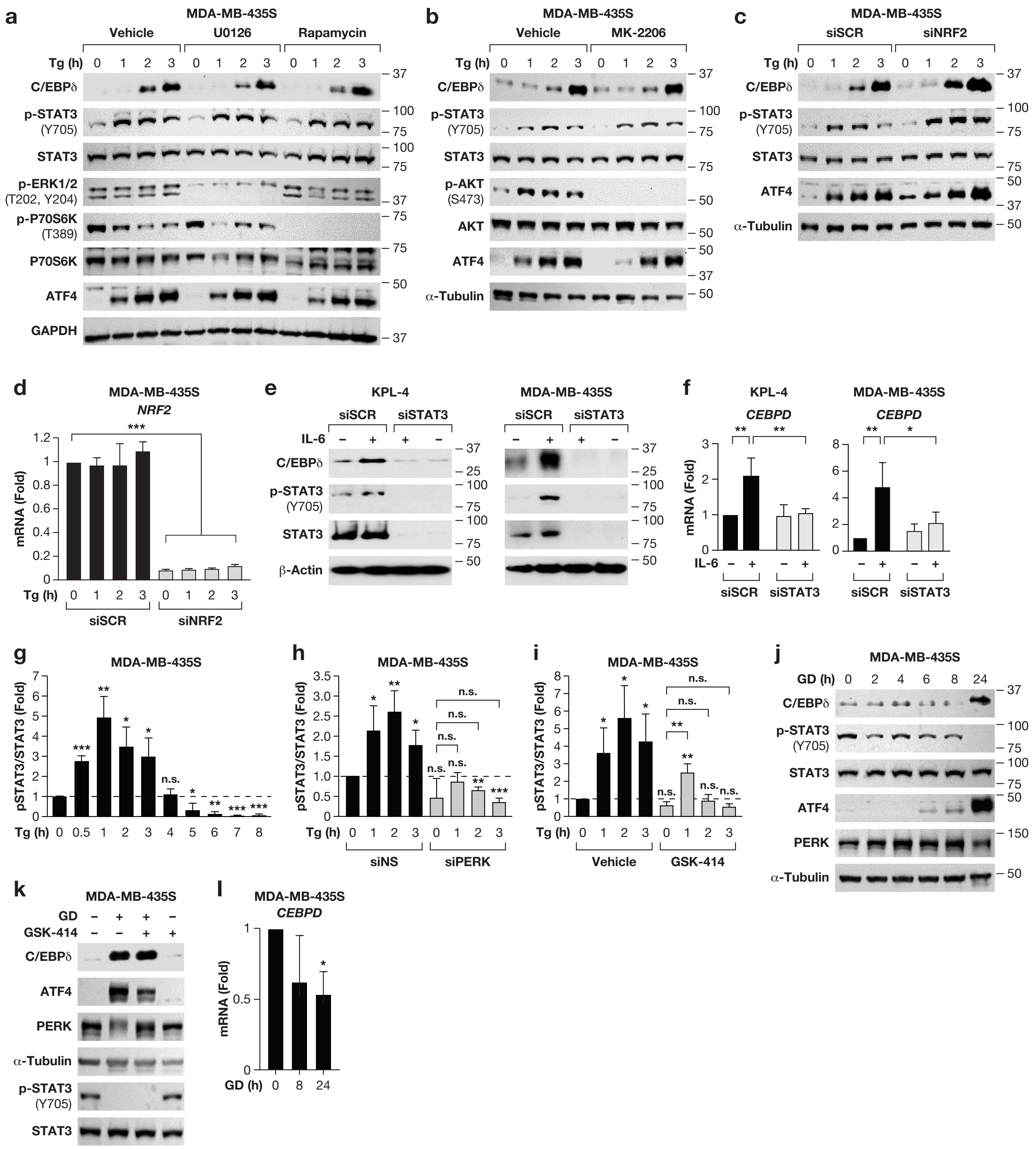

Supplemental Figure 3

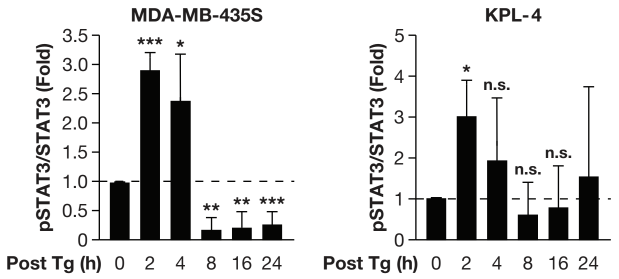

Supplemental Figure 4

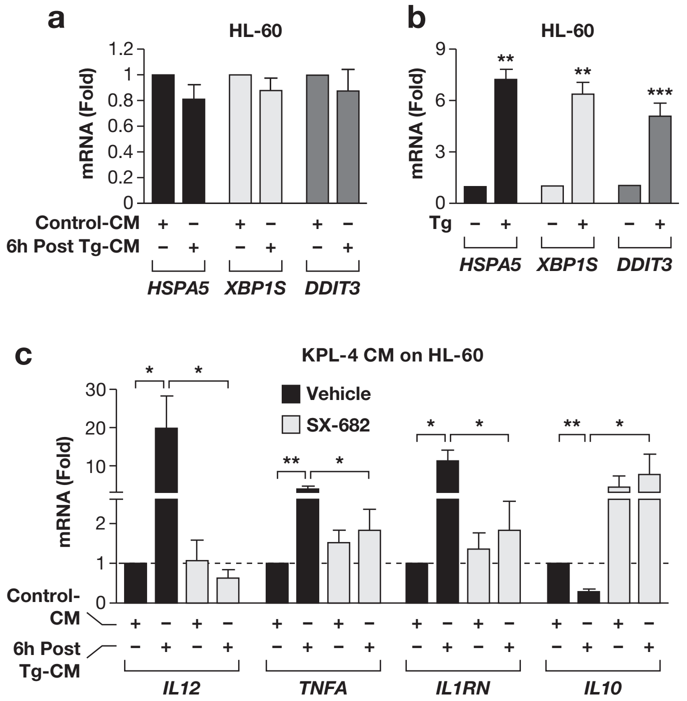

Supplemental Figure 5

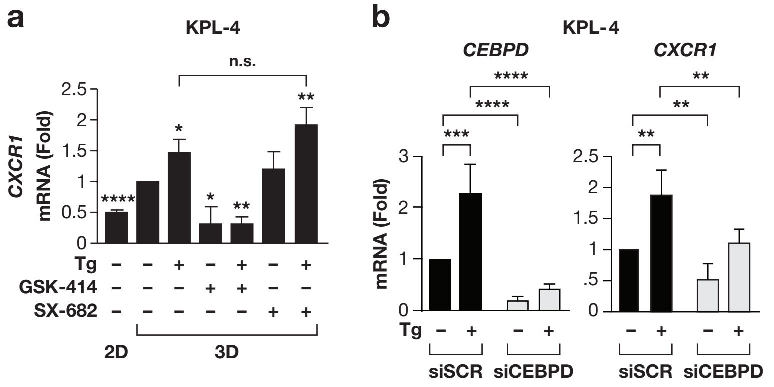

Supplemental Figure 6

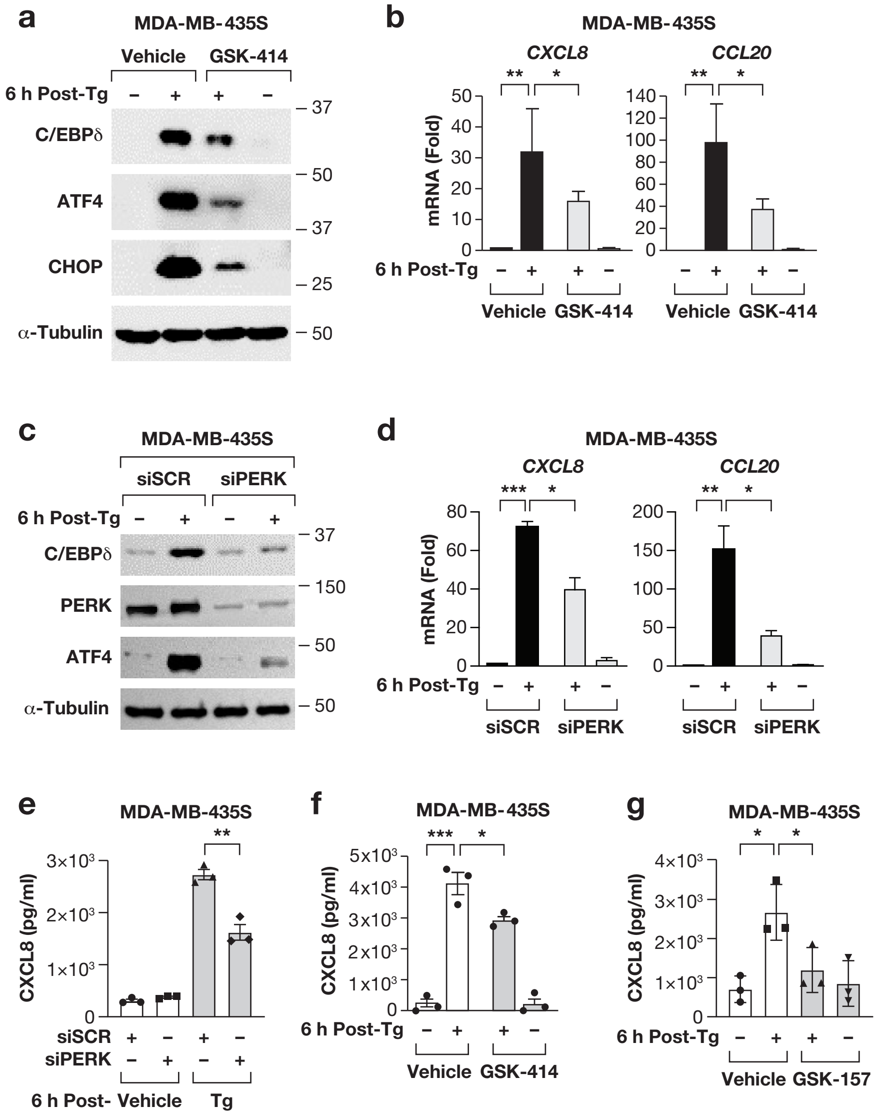

PERK signaling through C/EBPδ contributes to ER stress-induced expression of immunomodulatory and tumor promoting chemokines by cancer cells

Namratha Sheshadri, Dipak K. Poria, Shikha Sharan, Ying Hu, Chunhua Yan, Vishal N. Koparde, Kuppusamy Balamurugan, and Esta Sterneck

Table S3: Primers used for q-PCR

Gene Name	Sequence 5'-3' Forward	Sequence 5'-3' Reverse
ATF4	CTTGATGTCCCCCTTCGACC	GAAGGCATCCTCCTTGCTGT
ATF6	ACGGAGTATTTTGTCCGCCT	AGCAAAGAGAGCAGAATCCCAA
BIRC3	TCTGGGCAGCAGGTTTACAA	GCATTCTTTGGATAGTAAAACACCA
CCL20	CGAATCAGAAGCAGCAAGCAA	TTGCGCACACAGACAACTTT
CEBPD	CTGTCGGCTGAGAACGAGAA	TGAGGTATGGGTCGTTGCTG
CXCL8	GAGACAGCAGAGCACACAAG	GGCAAAACTGCACCTTCACAC
CXCR1	CTGATCTCTGACTGCAGCTCCT	CAGCAATGGTTTGATCTAACTGAAG
CXCR2	GGTCAGAAGTTTCATCGTCAAGG	TTAAATCCTGACTGGGTCGC
DDIT3	GCTGGAACCTGAGGAGAGAGTGTT	GCAGGATAATGGGGAGTGGCTGG
DNAJB9	GTCGGAGGGTGCAGGATATT	TTGATTTGGCGCTCTGATGC
EDEM1	CGCGGGGACCCTTCAAATC	AGGCTTCCCAGGACCCTTAT
EIF2AK3	CTCAGCGACGCGAGTACC	TGATAATTACTAATGACCTGCCGC
ERN1	TGCCTAGTCAGTTCTGCGTCC	GTCACTGTGCTGGTACTTCCAAA
GAPDH	AAGGTCGGAGTCAACGGATTTG	CCATGGGTGGAATCATATTGGAA
HSPA5	CACTCCTGAAGGGGAACGTC	TCAACCACCTTGAACGGCAA
IL21R	CTCTTTGGGAAGAGACGCCG	GTCTTGCCAGGTAAGGGTGAG
IRF1	ACCCTGGCTAGAGATGCAGA	TGCTTTGTATCGGCCTGTGT
NRF2	AGGTTGCCCACATTCCCAAA	AGTGACTGAAACGTAGCCGA
RPLP0	GCAATGTTGCCAGTGTCTGTC	GCCTTGACCTTTTCAGCAAGT
SREBF1	CTCCCTAGGAAGGGCCGTA	GCCGACTTCACCTTCGATGT
XBP1S	TGCTGAGTCCGCAGCAGGTG	GCTGGCAGGCTCTGGGGAAG
